# Dynamic simulation for reclaimed water reuse under multi-intervention policies in China

**DOI:** 10.1016/j.heliyon.2024.e25309

**Published:** 2024-02-01

**Authors:** Wei Wang, Fengping Wu

**Affiliations:** Business School, Hohai University, No.8, Fucheng West Road, Jiangning District, Nanjing, Jiangsu Province, 211100, China

**Keywords:** Water reuse, Price subsidy, Information disclosure supervision, Evolutionary game, Multi-agent-based model

## Abstract

Unconventional water constitutes the fundamental approach to addressing global water scarcity and achieving the sustainable circulation of water resources. Due to the significant environmental advantages and economical production costs, reclaimed water has emerged as a preeminent unconventional source. However, the use in China confronts the predicament of oversupply relative to demand, requiring policy measures to overcome this challenge. Limited research exists on the combined impact of subsidies and water quality information disclosure supervision on reclaimed water utilization, potentially underestimating the practical incentivizing role of water quality information disclosure. Therefore, based on the framework of 'external environment-perceived value-utilization intention,' a multi-agent-based simulation model driven by evolutionary game theory is constructed, from micro to macro perspective, to investigate the composite effects of subsidies and water quality information disclosure supervision on public intentions for reclaimed water utilization and the evolutionary track of public decision-making. The results showed that (1) The influence of subsidies on the public's inclination toward reclaimed water has regional heterogeneity. In regions with average economic development, the subsidy policy shows an inverted U-shaped correlation with the public's intention to reclaimed water, indicating the presence of an optimal value for maximizing the promotional effect of subsidies. Conversely, the effect is less discernible in regions with higher economic development. (2) In regions with average economic development, supervision of information disclosure behavior can avert the diminishing incentivizing effects under radical subsidies, but the assistance of various supervision intensities is different. (3) In regions with higher economic development, the incentive effect of subsidies can be positively modulated by the supervision policy. Interactions between subsidy and supervision policies evoke diverse chain reactions under varying intensities in these regions, and the combination of moderate subsidies and high supervision emerges as the most optimal strategy to advance reclaimed water development.

## 1 Introduction

In recent years, climate change, frequent human activities, and other factors have facilitated the global proliferation of drought. Diminishing river flows, lowered river water levels, desiccated wetlands, and worsening water quality all contribute to a worldwide water security situation that is far from optimistic. According to the UN World Water Development Report 2023, globally, 2 billion people do not have safe drinking water, 3.6 billion (46 %) lack access to safely managed sanitation, and between two and three billion people experience water shortages for at least one month per year. By the year 2050, it is projected that the urban population in cities grappling with global water scarcity will reach an estimated 170 to 240 million [1]. Of 17 United Nations Sustainable Development Goals, SDG6 is quite crucial, aiming to ensure universal access to water and sanitation facilities. Specifically, SDG6.3 targets significantly increasing wastewater recycling and safe reuse by 2030 to improve water quality and address water scarcity issues [2]. Confronting this challenge, in 2015, the Circular Economy Action Plan explicitly called for adopting water-saving policies centered on reclaimed water, encouraging member countries to enhance water efficiency [3]. In 2019, the United States implemented the National Water Reuse Action Plan to ensure the resilience of water resources [4]. Hence, it is evident that water reuse is an indispensable measure for sustainable development.

Water resources in China exhibit spatiotemporal disparities, with an escalating contradiction between supply and demand. This incongruity has precipitated water scarcity and pollution issues, presenting formidable impediments to socioeconomic advancement. Consequently, unconventional water sources have assumed a pivotal role in contemporary water resource security management and the pursuit of sustainable development [5,6]. According to the China Water Resources Bulletin [7], in 2022, China's total water supply and water consumption were both 5998.2 billion m^3^. Among them, unconventional water sources amounted to 175.8 billion m^3^, and reclaimed water accounts for approximately 85 % of non-conventional water sources. The Water Law of the People's Republic of China (2002 Revision) [8] has explicitly encouraged reclaimed water and wastewater recycling since 2002. Regrettably, despite a two-decade-long advocacy, the overall adoption of reclaimed water remains modest due to inhibiting factors, such as an imperfect pricing system, user aversion, and lack of trust [9]. Based on statistical data (from China urban-rural construction statistical yearbook 2022), by the end of 2022, nationwide reclaimed water utilization amounted to a mere 17.9 billion cubic meters. Furthermore, only Beijing, Hebei, Inner Mongolia, Henan, Guangdong, and Ningxia have attained the 2025 objectives stipulated in the Guideline Sets Goals for Wastewater Use (referred to as the "Pilot Program") [10]. Hence, the pressing predicament of limited progress in promoting reclaimed water utilization necessitates immediate resolution. Unfortunately, reclaimed water has yet to realize its full potential in China. This underutilization stems from multifaceted challenges. Firstly, the dual character of reclaimed water as both a commodity and a public good complicates the pricing system and puts it at a cost disadvantage compared to freshwater. Secondly, there's a conspicuous lack of public awareness regarding reclaimed water technology and negative stereotypes. In developing reclaimed water within a modern water management framework, adherence to 'market-driven, government-guided, and policy-oriented' is crucial [11,12]. Therefore, the quest for a reasonable government action model to augment the cost-effectiveness and mitigate public trust barriers is a pivotal path toward sizable utilization of reclaimed water, and a guarantee for implementing the most rigorous water resource management system.

Reclaimed water, derived from wastewater, is often met with user resistance, thus underscoring the vital role of government support in its early development [13,14]. And the formulation and implementation of policies require alignment with the impediments and drivers of reclaimed water development. The public's inclination toward reclaimed water hinges on the equilibrium between perceived benefits and perceived risks. Perceived risks stem from the origin [15], quality [16], and prevailing stereotypes [11], while providing product-specific information is a productive means to bolster confidence and counteract adverse preconceptions [17,18]. Hou et al. [19]found that reclaimed water information disclosure has the most significant impact on public water-saving awareness and acceptance. Fu and Liu [20]divided the informational intervention policies into demonstration-guided, knowledge-popularization, and environmental motivation driven. Through a single-category implicit association test, he found that demonstration-guided policies have the optimal effect on reducing the aversion. Based on the multi-agent model, Ding et al. [21]observed that demonstration-guided policies have a favorable impact on various forms of reclaimed water utilization behaviors. And the effects of knowledge-popularization and environmental motivation-driven policies will respectively rise and fall with increased exposure. The perceived benefit may stem from the economy of reclaimed water use, and in such cases, monetary incentives can serve as practical policy tools to mediate it [22]. Gao et al. [23]compared the impact of two mechanisms, direct subsidy and indirect subsidy, on the public's willingness to use reclaimed water. Wang et al. [9]found that the incentive effect of subsidy policies exhibits regional heterogeneity with varying initial levels of reclaimed water reuse.

The policy system represents a quintessential intricate adaptive structure, encompassing numerous stakeholders with diverse resource allocations and competencies during the phases of policy inception and execution [24].And the policy effects emerge as a complex interplay among various stakeholders. Agent-based model (ABM) is a standard tool extensively employed in public policy research, such as fiscal policy [25], monetary policy [26,27], resource management policy [28,29], and technology diffusion policy [[30], [31], [32]]. Moreover, this model can depict and deduct various macro-level phenomena by analyzing the micro-level causation, mechanisms, and evolutionary processes, facilitating the resolution of policy formulation, implementation, and evaluation challenges [33].

The existing literature serves as the theoretical basis and model reference for us. Unfortunately, there are still some deficiencies. On the one hand, compared with the government authorities, reclaimed water enterprises enjoy an information edge. Their decision to disclose product information significantly impacts public awareness. However, research on selecting the regulatory of water quality information disclosure as a policy tool is rare. In addition, few scholars have applied ABM to the simulation of reclaimed water policy. Ding et al. [21]used ABM to investigate informative reclaimed water policies. Yet their model exclusively focused on the public group, omitting interactions between diverse groups, which limited the examination of intergroup dynamics.

Hence, our research questions (RQ) are as follows.

RQ1 How do subsidy policies impact the public's willingness to utilize reclaimed water?

RQ2 How does the combination of water quality information disclosure supervision and subsidy policies affect the public's willingness to utilize reclaimed water?

RQ3 Is there heterogeneity in the policy effects across distinct regions?

The main contributions of this study are as follows: (1) A comprehensive framework for reclaimed water utilization was developed based on ‘external environment-perceived value-utilization intention.’ Within this framework, price subsidies and water quality information disclosure supervision were chosen as policy tools to enhance the perceived benefits and mitigate perceived risks. (2) The multi-agent-based model driven by evolutionary game theory was built, and this model underwent a two-step realization. Initially, a tripartite evolutionary game model was established to analyze the interaction dynamics among three groups. Subsequently, the multi-agent-based model grounded in evolutionary game theory was built, from a micro to macro perspective, to delve into the mechanisms of subsidies and supervision on reclaimed water use and the evolutionary track of public decision-making. (3) The rational random perturbations have been introduced into the multi-agent simulation model to ensure that the model further aligns with the complexity of the strategy selection process in the real world.

The remainder of the paper is organized as follows. Section 2 introduces the model in this paper. Section 3 and Section 4 describe the simulation results and scenario analysis. Section 5 presents conclusions and policy implications.

## 2 Methodology

### 2.1 Research framework

**Fig. 1:**
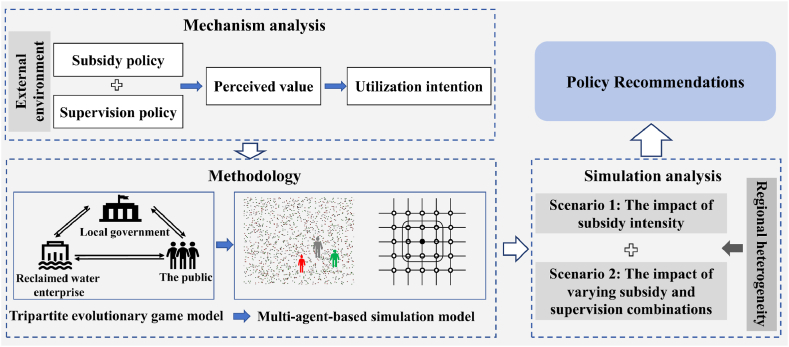
The framework of the research system.

In this research, we initially construct a framework of ‘external environment - perceived value - utilization intention’ and employ price subsidies and water quality information disclosure supervision as policy instruments to dissect the mechanisms of public reclaimed water utilization under these two policies. On this basis, we develop a multi-agent-based model driven by evolutionary game theory, incorporating local government, reclaimed water enterprises, and the public as stakeholders. Through simulation analysis, we investigated the public's willingness to utilize reclaimed water under varying intensities of subsidies and supervision policies in regions characterized by different levels of economic development. This endeavor aims to furnish comprehensive insights into the specific management of reclaimed water. The research framework is shown in Fig. 1.

### 2.2 The mechanism of reclaimed water utilization under policy regulation

In order to alleviate the pressure of water resource scarcity and ensure sustainable use of water resources, China adheres to a hybrid approach, combining government leadership with market means. Reclaimed water has substantial ecological and environmental benefits, making it impractical to rely on market mechanisms predominantly for allocation. Therefore, government involvement is essential. We developed a comprehensive framework based on ‘external environment-perceived value-utilization intention’ to explore the mechanism of reclaimed water utilization under policy regulation. In the ‘external environment-perceived value-utilization intention’ framework, the external environment, including the price and quality of reclaimed water, influences public perception in two key aspects: perceived benefits and perceived risks. These perceptions shape the overall perceived value of reclaimed water, directly affecting the public's willingness to adopt it.

**Fig. 2:**
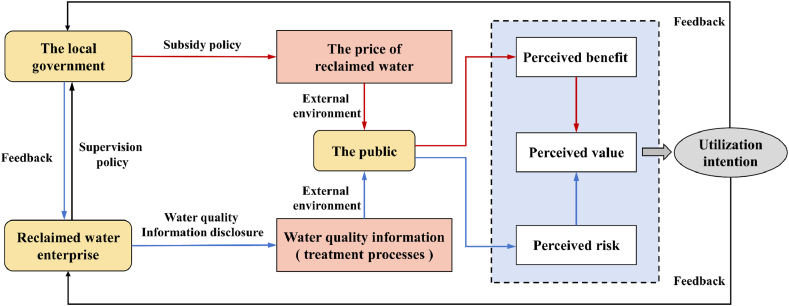
The mechanism of reclaimed water utilization under the framework of 'external environment-perceived value-utilization intention’.

As shown in Fig. 2, whether the public is willing to use reclaimed water depends on perceived value, divided into perceived benefits and risks. Perceived risk is an essential variable in behavioral research. It refers to an individual's apprehension of uncertainty or unfavorable consequences linked to a specific entity, and its intensity is contingent upon the possibility of the risks and the potential magnitude of resulting losses [34]. We defined perceived risk as the extent to which the public is exposed to health threats when utilizing reclaimed water. Perceived benefit refers to an individual's affirmation of favorable outcomes from the adoption action [35]. We defined perceived benefit as the extent to which the public perceives the potential advantages of reclaimed water use, such as reducing usage costs. Typically, there is a negative correlation between perceived risk and the public's choice of reclaimed water technologies, while it is also inversely related to perceived benefits [36,37].

Reclaimed water can create new supply and reduce water pollution [13]. Its implementation on a global scale, particularly in the United States, Japan, Australia, and Singapore, has demonstrated significant economic and environmental benefits [[38], [39], [40], [41]] [[38], [39], [40], [41]] [[38], [39], [40], [41]]. However, the development process of reclaimed water in China is still at an early stage. Hence, governmental engagement is considered imperative. Related policies are implemented on both the supply and demand sides to bolster public acceptance directly or indirectly. On the one hand, the price subsidies on the demand side effectively magnify perceived benefits by lowering the purchasing cost of reclaimed water. Simultaneously, supervision of water quality information disclosure at the supply side mitigates perceived risks by increasing customer mastery of water quality information. Nevertheless, under supervision, some enterprises may still engage in speculative practices by falsely disclosing water quality information to boost transaction volumes for profit. While this behavior can bring additional income, once discovered, there is a risk of punishment.

### 2.3 Tripartite evolutionary game model

#### 2.3.1 Model assumptions and parameter settings

We made the following assumptions to simplify the model without changing the nature of the critical problem. The notations involved in the model and their meanings are given in Table 1.

(1) In a reclaimed water utilization system composed of the local governments, reclaimed water enterprises, and the public, comprehensive information cannot be obtained due to the inability to ascertain the evolutionary object's selection preferences and profit variations. Therefore, the information of the stakeholders in the game is not entirely symmetrical.
(2) Each player has two strategy choices. The strategy sets of the local governments are {*PR*, *NR*}, denoting positive and negative regulation, respectively. Strategy sets of reclaimed water enterprises are {*DI*, *CI*}, indicating water quality information disclosure and concealment. Similarly, strategy sets of the public are {*FW*, *RW*}, denoting fresh water and reclaimed water. The probability of choosing the strategy PR among government groups, strategy DI among enterprise groups, strategy RW are x,y,z, respectively, and x,y,z∈[0,1].
(3) Reclaimed water use holds social benefits and public welfare. Government-led regulatory services and complementary support are critical to improving the economic operation of sewage reuse. Positive regulation cost is $C_{g}$, and the government's affordability is ε. Under this strategic choice, the local governments can achieve comprehensive benefits ${CB}_{g}$. When the local governments opt for negative regulation, they can achieve additional leisure benefits $EB_{g}$ but may face penalties from higher authorities $P_{g}$.
(4) Information disclosure serves as a communication bridge between enterprises and various stakeholders in the external environment. The benefits derived from full information disclosure are $B_{e}$ (encompassing enhancements in reputation, fundraising capacity, and a reduction in fundraising costs), but this comes with the cost $C_{e}$. Simultaneously, there is an associated government reward $\alpha R_{e}$ (where α represents the reward coefficient). As independent economic men, enterprises may be engaged in self-serving behaviors through information asymmetry. In such cases, information concealment can lead to speculative gains $H_{e}$, but once discovered, it may result in penalties $\gamma P_{e}$ (where γ is the punishment coefficient).
(5) When the public uses reclaimed water under information disclosure, it can bring cost savings $S_{p}$. However, in cases of information concealment, reclaimed water utilization may lead to losses $L_{p}$ for the public (assuming that there are hidden dangers such as substandard effluent quality when concealing information). Fortunately, the public may be entitled to compensation $B_{p}$ ($B_{p}\geq L_{p}$) from enterprises under the positive regulation. Also, the local governments will provide price subsidies $\beta R_{p}$ (β is the subsidy intensity) to the public who actively uses reclaimed water.

**Table 1:** Notations and descriptions.

**Notions**	**Meaning**
**Abbreviations**	
*PR*, *NR*	Strategy positive and negative regulation
*DI*, *CI*	Strategy water quality information disclosure and concealment
*FW*, *RW*	Strategy fresh water and reclaimed water
**Parameters**	
$C_{g}$	The cost of strategy PR
${CB}_{g}$	Comprehensive benefits under strategy PR
$EB_{g}$	Additional leisure benefits under strategy NR
$P_{g}$	Penalties from higher authorities under strategy NR
$B_{e}$	The benefits derived from full information disclosure
$C_{e}$	The cost of strategy DI
$\alpha R_{e}$	Government reward under strategy DI, α is the reward coefficient
$H_{e}$	Speculative gains under strategy CI
$\gamma P_{e}$	Penalties under strategy CI, γ is the punishment coefficient
$S_{p}$	Cost savings under strategy RW
$L_{p}$	Losses with using reclaimed water with substandard effluent quality
$B_{p}$	Compensation from enterprises when suffer losses
$\beta R_{p}$	Price subsidies, β is the subsidy intensity
x,y,z	The probability of governments choosing PR, enterprises choosing DI, the public choosing RW
$E_{g1}$, $E_{g2}$, $\bar{E_{g}}$	The expected profits of strategy PR and NR, and the average expected profits of these two choices
$E_{e1}$, $E_{e2}$, $\bar{E_{e}}$	The expected profits of strategy DI and CI, and the average expected profits of two strategies
$E_{p1}$, $E_{p2}$, $\bar{E_{p}}$	The expected profits of strategy RW and FW, and the average expected profits of two strategies
$A_{G\left(i\right)}$, $A_{E\left(j\right)}\text{,}$$A_{p\left(k\right)}$	The ith government agent, the jth reclaimed water agent, and the kth public agent
$E_{i}^{\mathrm{PR}}\left(t\right),E_{i}^{NR}\left(t\right)$	The expected benefits of $A_{G\left(i\right)}$ with strategy PR and NR
$E_{j}^{DI}\left(t\right)$, $E_{j}^{CI}\left(t\right)$	The expected benefits of $A_{E\left(j\right)}$ with strategy DI and CI
$E_{k}^{RW}\left(t\right)$, $E_{k}^{FW}\left(t\right)$	The expected benefits of $A_{p\left(k\right)}$ with strategy RW and FW
ϑ,μ,σ	Error terms

#### 2.3.2 Evolutionary model construction

Based on the above assumptions, the payment matrix of three players is shown in Table 2.

**Table 2:** The payoff matrix for three players.

	PR (*z*)		NR (*1-z*)	
	DI (*y*)	CI (*1-y*)	DI (*y*)	CI (*1-y*)
**RW (*z*)**	$CB_{g}-{\varepsilon C}_{g}-\alpha R_{e}-\beta R_{p}$	$CB_{g}-{\varepsilon C}_{g}+\gamma P_{e}$	$EB_{g}-P_{g}-\alpha R_{e}-\beta R_{p}$	$EB_{g}-P_{g}-\beta R_{p}$
	$B_{e}+\alpha R_{e}-C_{e}$	$H_{e}-\gamma P_{e}-B_{p}$	$B_{e}+\alpha R_{e}-C_{e}$	$H_{e}$
	$S_{p}+\beta R_{p}$	$S_{p}-L_{p}+\beta R_{p}+B_{p}$	$S_{p}+\beta R_{p}$	$S_{p}-L_{p}+\beta R_{p}$
**FW (*1-z*)**	$CB_{g}-{\varepsilon C}_{g}-\alpha R_{e}$	$CB_{g}-{\varepsilon C}_{g}+\gamma P_{e}$	$EB_{g}-P_{g}-\alpha R_{e}$	$EB_{g}-P_{g}$
	$B_{e}+\alpha R_{e}-C_{e}$	$H_{e}-\gamma P_{e}$	$B_{e}+\alpha R_{e}-C_{e}$	$H_{e}$
	0	0	0	0

According to Table 1, $E_{g1}$ and $E_{g2}$ represent the expected profits of strategy PR and NR, respectively. And $\bar{E_{g}}$ is the average expected profits of these two choices. The expected profits of the local government are shown in equations (1), (3).

(1) $$\begin{array}{c} E_{g1}=yz\left({CB}_{g}-{\varepsilon C}_{g}-\alpha R_{e}-\beta R_{p}\right)+y\left(1-z\right)\left({CB}_{g}-{\varepsilon C}_{g}-\alpha R_{e}\right)+\\ \;\left(1-y\right)z\left({CB}_{g}-{\varepsilon C}_{g}+\gamma P_{e}-\beta R_{p}\right)+\left(1-y\right)\left(1-z\right)\left({CB}_{g}-{\varepsilon C}_{g}+\gamma P_{e}\right) \end{array}$$

(2) $$\begin{array}{c} E_{g2}=yz\left(EB_{g}-P_{g}-\alpha R_{e}-\beta R_{p}\right)+y\left(1-z\right)\left(EB_{g}-P_{g}-\alpha R_{e}\right)+\\ \left(1-y\right)z\left(EB_{g}-P_{g}-\beta R_{p}\right)+\left(1-y\right)\left(1-z\right)\left(EB_{g}-P_{g}\right) \end{array}$$

(3) $$\begin{array}{c} \bar{E_{g}}=xE_{g1}+\left(1-x\right)E_{g2} \end{array}$$

$E_{e1}$ and $E_{e2}$ are the expected profits of strategy DI and CI. And $\bar{E_{e}}$ indicates the average expected profits of two strategies. The expected profits of the reclaimed water enterprises are shown in equations (4), (5), (6).

(4) $$\begin{array}{c} E_{e1}=xz\left(B_{e}+\alpha R_{e}-C_{e}\right)+x\left(1-z\right)\left(B_{e}+\alpha R_{e}-C_{e}\right)+\left(1-x\right)z\left(B_{e}+\alpha R_{e}-C_{e}\right)+\\ \left(1-x\right)\left(1-z\right)\left(B_{e}+\alpha R_{e}-C_{e}\right) \end{array}$$

(5) $$\begin{array}{c} E_{e2}=xz\left(H_{e}-\gamma P_{e}-B_{p}\right)+x\left(1-z\right)\left(H_{e}-\gamma P_{e}\right)+\left(1-x\right)zH_{e}+\left(1-x\right)\left(1-z\right)H_{e} \end{array}$$

(6) $$\begin{array}{c} \bar{E_{e}}=yE_{e1}+\left(1-y\right)E_{e2} \end{array}$$

Similarly, $E_{p1}$ and $E_{p2}$ represent the expected profits of strategy RW and FW. And $\bar{E_{p}}$ indicates the average expected profits of two strategies. The expected profits of the public are shown in equations (7), (8), (9).

(7) $$\begin{array}{c} E_{p1}=xy\left(S_{p}+\beta R_{p}\right)+x\left(1-y\right)\left(S_{p}-L_{p}+\beta R_{p}+B_{p}\right)+\left(1-x\right)y\left(S_{p}+\beta R_{p}\right)+\\ \left(1-x\right)\left(1-y\right)\left(S_{p}-L_{p}+\beta R_{p}\right) \end{array}$$

(8) $$\begin{array}{c} E_{p2}=0 \end{array}$$

(9) $$\begin{array}{c} \bar{E_{p}}=zE_{p1}+\left(1-z\right)E_{p2} \end{array}$$

#### 2.3.3 Equilibrium strategy stability analysis

Guided by the principles of replicator dynamics in evolutionary game theory [42] and integrating the expected profits model, dynamic replicator equations for various groups with strategy PR, strategy CI, and strategy RW are shown in equation (10), (11), (12).

(10) $$\begin{array}{c} F\left(x\right)=\frac{dx}{dt}=x\left(1-x\right)\left(-y\gamma P_{e}+{CB}_{g}-\varepsilon C_{g}+\gamma P_{e}-B_{e}+P_{g}\right) \end{array}$$

(11) $$\begin{array}{c} F\left(y\right)=\frac{dy}{dt}=y\left(1-y\right)\left(x\gamma P_{e}+xzB_{p}+B_{e}+\alpha R_{e}-C_{e}-H_{e}\right) \end{array}$$

(12) $$\begin{array}{c} F\left(z\right)=\frac{dz}{dt}=z\left(1-z\right)\left(xB_{p}+yL_{p}-xyB_{p}+S_{p}-L_{p}+\beta R_{p}\right) \end{array}$$

Let F(x)=0， F(y)=0， F(z)=0, 9, and internal equilibrium points of system are obtained, that are $E_{1}\left(0,0,0\right)$， $E_{2}\left(0,0,1\right)$， $E_{3}\left(0,1,0\right)$， $E_{4}\left(1,0,0\right)$， $E_{5}\left(1,1,0\right)$， $E_{6}\left(1,0,1\right)$， $E_{7}\left(0,1,1\right)$， $E_{8}\left(1,1,1\right)$, $E_{9}\left(x*,y*,z*\right)$. The evolutionarily stable strategy (EES) depicts the steady at an equilibrium point. In multi-population asymmetric evolutionary games, the equilibrium is necessarily a strict Nash equilibrium [42], and its stability can only be achieved with pure strategies [43]. Therefore, in the evolutionary game of reclaimed water utilization, we discussed the stability of these equilibrium points, except for $E_{9}\left(x*,y*,z*\right)$.

The Lyapunov indirect method is employed to assess the evolutionary stable strategies of a system. The criteria for assessment are as follows: when all the real parts of the eigenvalues of the Jacobian matrix at a specific equilibrium point are negative, the system achieves an asymptotically stable state at that equilibrium point. Conversely, if there is at least one eigenvalue with a real part of zero or a positive value, the system is unstable at that equilibrium point. The non-linear system composed of the government, reclaimed water enterprises and the public is linearized to assess the local stability of the corresponding Jacobian matrix [44]. The Jacobian matrix (13)–(14) are as follows.

(13) $$\begin{array}{c} J=\left[\begin{array}{ccc} \frac{\partial F\left(x\right)}{\partial x} & \frac{\partial F\left(x\right)}{\partial y} & \frac{\partial F\left(x\right)}{\partial z}\\ \frac{\partial F\left(y\right)}{\partial x} & \frac{\partial F\left(y\right)}{\partial y} & \frac{\partial F\left(y\right)}{\partial z}\\ \frac{\partial F\left(z\right)}{\partial x} & \frac{\partial F\left(z\right)}{\partial y} & \frac{\partial F\left(z\right)}{\partial z} \end{array}\right] \end{array}$$

$$=\begin{array}{c} \left[\begin{array}{ccc} \left(1-2x\right)\left(\begin{array}{c} -y\gamma P_{e}+{CB}_{g}-{\varepsilon C}_{g}\\ +\gamma P_{e}-{EB}_{g}+P_{g} \end{array}\right) & x\left(1-x\right)\left(-\gamma P_{e}\right) & 0\\ y\left(1-y\right)\left(\gamma P_{e}+zB_{p}\right) & \left(1-2y\right)\left(\begin{array}{c} x\gamma P_{e}+xzB_{p}+B_{e}\\ +\alpha R_{e}-C_{e}-H_{e} \end{array}\right) & y\left(1-y\right)xB_{p}\\ z\left(1-z\right)\left(1-y\right)B_{p} & z\left(1-z\right)\left(L_{p}-xB_{p}\right) & \left(1-2z\right)\left(\begin{array}{c} xB_{p}+yL_{p}-xyB_{p}\\ +S_{p}-L_{p}+\beta R_{p} \end{array}\right) \end{array}\right] \end{array}$$

The Jacobian matrix at point $E_{1}\left(0,0,0\right)$ is presented as follows.

(14) $$\begin{array}{c} J_{1}=\left[\begin{array}{ccc} \begin{array}{c} R_{g}-\varepsilon C_{g}+\gamma P_{e}-EB_{g}+P_{g} \end{array} & 0 & 0\\ 0 & \begin{array}{c} B_{e}+\alpha R_{e}-C_{e}-H_{e} \end{array} & 0\\ 0 & 0 & S_{p}-L_{p}+\beta R_{p} \end{array}\right] \end{array}$$

The eigenvalues of $J_{1}$ are $\lambda_{1}=R_{g}-{\varepsilon C}_{g}+\gamma P_{e}-EB_{g}+P_{g}$， $\lambda_{2}=B_{e}+\alpha R_{e}-C_{e}-H_{e}$， $\lambda_{3}=S_{p}-L_{p}+\beta R_{p}$. Similarly, the eigenvalues of the other 7 equilibrium points can be obtained, as shown in Table 3.

**Table 3:** Eigenvalue of each equilibrium point.

Point	$\lambda_{1}$	$\lambda_{2}$	$\lambda_{3}$
$E_{1}\left(0,0,0\right)$	$\left({CB}_{g}-{\varepsilon C}_{g}+\gamma P_{e}\right)-\left(EB_{g}-P_{g}\right)$	$\left(B_{e}+\alpha R_{e}-C_{e}\right)-H_{e}$	$S_{p}+\beta R_{p}-L_{p}$
$E_{2}\left(0,0,1\right)$	$\left({CB}_{g}-{\varepsilon C}_{g}+\gamma P_{e}\right)-\left(EB_{g}-P_{g}\right)$	$\left(B_{e}+\alpha R_{e}-C_{e}\right)-H_{e}$	$-\left(S_{p}+\beta R_{p}-L_{p}\right)$
$E_{3}\left(0,1,0\right)$	$\left({CB}_{g}-\varepsilon C_{g}\right)-\left(EB_{g}-P_{g}\right)$	$-\left[\left(B_{e}+\alpha R_{e}-C_{e}\right)-H_{e}\right]$	$S_{p}+\beta R_{p}$
$E_{4}\left(1,0,0\right)$	$-\left[\left({CB}_{g}-{\varepsilon C}_{g}+\gamma P_{e})-(EB_{g}-P_{g}\right)\right]$	$\left(B_{e}+\alpha R_{e}-C_{e}\right)-\left(H_{e}-\gamma P_{e}\right)$	$S_{p}+\beta R_{p}+B_{p}-L_{p}$
$E_{5}\left(1,1,0\right)$	$-\left[\left({CB}_{g}-{\varepsilon C}_{g}\right)-\left(EB_{g}-P_{g}\right)\right]$	$-\left[\left(B_{e}+\alpha R_{e}-C_{e}\right)-\left(H_{e}-\gamma P_{e}\right)\right]$	$S_{p}+\beta R_{p}$
$E_{6}\left(1,0,1\right)$	$-\left[\left({CB}_{g}-{\varepsilon C}_{g}+\gamma P_{e})-(EB_{g}-P_{g}\right)\right]$	$\left(B_{e}+\alpha R_{e}-C_{e}\right)-\left(H_{e}-\gamma P_{e}-B_{p}\right)$	$-\left(S_{p}+\beta R_{p}+B_{p}-L_{p}\right)$
$E_{7}\left(0,1,1\right)$	$\left({CB}_{g}-{\varepsilon C}_{g}\right)-\left(EB_{g}-P_{g}\right)$	$-\left[\left(B_{e}+\alpha R_{e}-C_{e}\right)-H_{e}\right]$	$-\left(S_{p}+\beta R_{p}\right)$
$E_{8}\left(1,1,1\right)$	$-\left[\left({CB}_{g}-\varepsilon C_{g}\right)-\left(EB_{g}-P_{g}\right)\right]$	$-\left[\left(B_{e}+\alpha R_{e}-C_{e}\right)-\left(H_{e}-\gamma P_{e}-B_{p}\right)\right]$	$-\left(S_{p}+\beta R_{p}\right)$

Actually, various factors influence the strategic choices of all participants. By analyzing the equilibrium points above, we have obtained EES under different scenarios (Table 4) and subsequently analyzed the practical significance of these stable points.

(1) Scenario 1: When $\left({CB}_{g}-{\varepsilon C}_{g}+\gamma P_{e}\right)<\left(EB_{g}-P_{g}\right)$, $B_{e}+\alpha R_{e}-C_{e}<H_{e}$, $S_{p}+\beta R_{p}<L_{p}$, sum of the strategy PR profit and the penalties levied on enterprises is less than that of strategy NR, the strategy DI profit is less than speculative gains, and the risk from purchasing subpar reclaimed water is less than 0. For $E_{1}\left(0,0,0\right)$, regulations and standards in the reclaimed water industry remain unclear, and the development of wastewater treatment plants is disorderly, with substantial investments and minimal returns. These elevate the risk of using reclaimed water. Hence, the stability in scenario 1 should be avoided.
(2) Scenario 2: When $\left({CB}_{g}-\varepsilon C_{g}\right)>\left(EB_{g}-P_{g}\right)$, $\left(B_{e}+\alpha R_{e}-C_{e}\right)<\left(H_{e}-\gamma P_{e}-B_{p}\right)$, sum of the strategy PR profit and the penalties levied on enterprises exceeds that of strategy NR, and the strategy DI profit is less than speculative gains. For $E_{1}\left(1,0,1\right)$, by leveraging the information advantage, reclaimed water enterprises can attain higher risk returns. Driven by arbitrage motives, strategy CI is dominant. However, the intensification of information asymmetry can undermine investor enthusiasm and even harm their own interests. Consequently, a stable state in scenario 2 is detrimental to the health of the reclaimed water market.
(3) Scenario 3: When $\left({CB}_{g}-{\varepsilon C}_{g}\right)>\left(EB_{g}-P_{g}\right)$, $\left(B_{e}+\alpha R_{e}-C_{e}\right)>\left(H_{e}-\gamma P_{e}-B_{p}\right)$, sum of the strategy PR profit and the penalties levied on enterprises exceeds that of strategy NR, and the strategy DI profit exceeds speculative gains. For $E_{1}\left(1,1,1\right)$, the regulation and management system of reclaimed water resources is relatively perfect, thereby reducing market disruption from incomplete contracts and facilitating trading trust establishment. Consequently, stability in scenario 3 is ideal for reclaimed water development. Currently, how to achieve the stage objectives by implementing multidimensional policy measures is one of the pivotal focal points in wastewater resource research.

**Table 4:** Stability at equilibrium point under different scenarios.

Scenarios	Equilibrium point	Stability condition
Scenario 1	$E_{1}\left(0,0,0\right)$	$\left({CB}_{g}-{\varepsilon C}_{g}+\gamma P_{e}\right)<\left(EB_{g}-P_{g}\right)$ $B_{e}+\alpha R_{e}-C_{e}<H_{e}$ $S_{p}+\beta R_{p}<L_{p}$
Scenario 2	$E_{2}\left(1,0,1\right)$	$\left({CB}_{g}-\varepsilon C_{g}\right)>\left(EB_{g}-P_{g}\right)$ $\left(B_{e}+\alpha R_{e}-C_{e}\right)<\left(H_{e}-\gamma P_{e}-B_{p}\right)$
Scenario 3	$E_{8}\left(1,1,1\right)$	$\left({CB}_{g}-\varepsilon C_{g}\right)>\left(EB_{g}-P_{g}\right)$ $\left(B_{e}+\alpha R_{e}-C_{e}\right)>\left(H_{e}-\gamma P_{e}-B_{p}\right)$

### 2.4 Multi-agent-based simulation model

The reclaimed water utilization system involves intricate interactions among economic, social, and ecological elements. Various stakeholders in this system have their own distinct goals while being deeply entrenched in specific social relationships. Moreover, they continuously learn and adapt their strategies throughout complex interactive processes, eventually contributing to the system's adaptation and evolution. However, the tripartite evolutionary game model is constructed to explore the evolution paths of strategic choices from a macro perspective, and this method may ignore the inherent cognitive limitations of individuals in complex real-world economic environments, leading to biases in system assessment. Therefore, this section built a multi-agent-based simulation model driven by evolutionary game theory to simulate the interaction between various agents, from micro to macro perspective, to delve into the mechanisms of different policies on reclaimed water use and the evolutionary track of public decision-making.

#### 2.4.1 Interactive logic design

The multi-agent-based simulation model we constructed is driven by evolutionary game rules, aiming to quantitatively study how decisions and mechanisms at the micro level lead to specific dynamic macro-level phenomena. Assuming that all agents in the game are on a two-dimensional square lattice with a side length of L, which satisfies periodic boundary conditions [33]. To enhance the algorithm's global search ability, we adopted a Moore type neighbor structure.

**Fig. 3:**
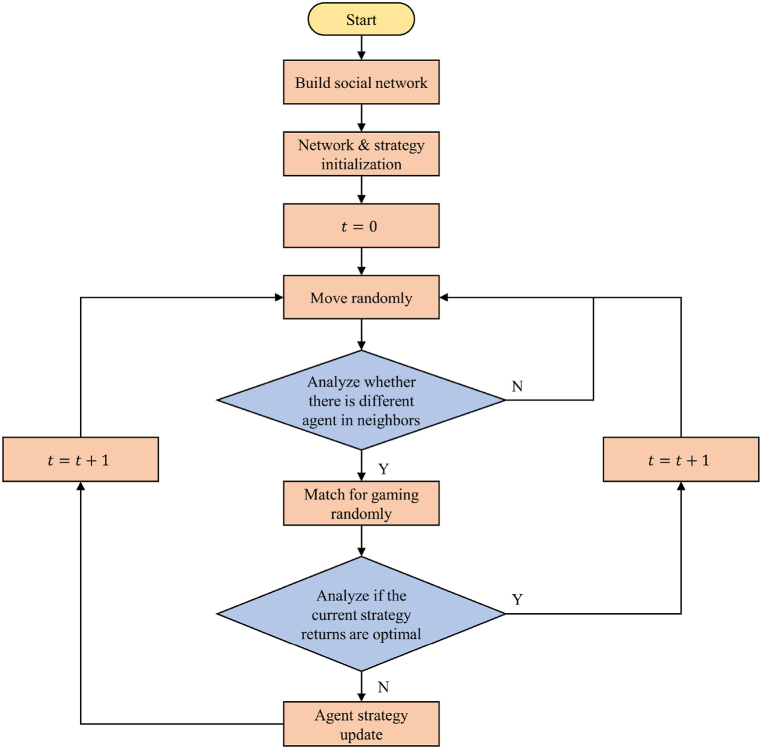
The interactive logic of agents.

The interactive mechanism for the agents is illustrated in Fig. 3. Initially, agents from three groups are randomly distributed across a two-dimensional square lattice. Within the neighborhood structure, the central agent needs to observe if there are any other agents present at neighboring locations. If no agent is found or all agents belong to the same group, the central agent will change its position randomly in the subsequent step. However, when agents from different groups are present, based on the inevitable interaction mechanism, the central agent pairs up randomly with one of its neighbors for a game, and assesses its game income compared to the expected benefits to decide whether to update the current strategy. Subsequently, it moves to a neighboring location in the next step for further interaction.

#### 2.4.2 Rule design

According to equation (1), (2), (4), (5), (7), and (8), we can get the expected benefits of the agent as follows.

The expected benefits of $A_{G\left(i\right)}$ are shown in equation (15), (16).

(15) $$E_{i}^{\mathrm{PR}}\left(t\right)=y\left(t\right)z\left(t\right)\left({CB}_{g}-{\varepsilon C}_{g}-\alpha R_{e}-\beta R_{p}\right)+y\left(t\right)\left[1-z\left(t\right)\right]\left({CB}_{g}-{\varepsilon C}_{g}-\alpha R_{e}\right)+\left[1-y\left(t\right)\right]z\left(t\right)\left({CB}_{g}-{\varepsilon C}_{g}+\gamma P_{e}-\beta R_{p}\right)+\left[1-y\left(t\right)\right]\left[1-z\left(t\right)\right]\left({CB}_{g}-{\varepsilon C}_{g}+\gamma P_{e}\right)$$

(16) $$E_{i}^{NR}\left(t\right)=y\left(t\right)z\left(t\right)\left(EB_{g}-P_{g}-\alpha R_{e}-\beta R_{p}\right)+y\left(t\right)\left[1-z\left(t\right)\right]\left(EB_{g}-P_{g}-\alpha R_{e}\right)+\left[1-y\left(t\right)\right]z\left(t\right)\left(EB_{g}-P_{g}-\beta R_{p}\right)+\left[1-y\left(t\right)\right]\left[1-z\left(t\right)\right]\left(EB_{g}-P_{g}\right)$$

The expected benefits of $A_{E\left(j\right)}$ are shown in equation (17), (18).

(17) $$E_{j}^{DI}\left(t\right)=x\left(t\right)z\left(t\right)\left(B_{e}+\alpha R_{e}-C_{e}\right)+x\left(t\right)\left[1-z\left(t\right)\right]\left(B_{e}+\alpha R_{e}-C_{e}\right)+\left[1-x\left(t\right)\right]z\left(t\right)\left(B_{e}+\alpha R_{e}-C_{e}\right)+\left[1-x\left(t\right)\right]\left[1-z\left(t\right)\right]\left(B_{e}+\alpha R_{e}-C_{e}\right)$$

(18) $$E_{j}^{CI}\left(t\right)=x\left(t\right)z\left(t\right)\left(H_{e}-\gamma P_{e}-B_{p}\right)+x\left(t\right)\left[1-z\left(t\right)\right]\left(H_{e}-\gamma P_{e}\right)+\left[1-x\left(t\right)\right]z\left(t\right)H_{e}+\left[1-x\left(t\right)\right]\left[1-z\left(t\right)\right]H_{e}$$

The expected benefits of $A_{p\left(k\right)}$ are shown in equation (19), (20).

(19) $$E_{k}^{RW}\left(t\right)=x\left(t\right)y\left(t\right)\left(S_{p}+\beta R_{p}\right)+x\left(t\right)\left[1-y\left(t\right)\right]\left(S_{p}-L_{p}+\beta R_{p}+B_{p}\right)+\left[1-x\left(t\right)\right]y\left(t\right)\left(S_{p}+\beta R_{p}\right)+\left[1-x\left(t\right)\right]\left[1-y\left(t\right)\right]\left(S_{p}-L_{p}+\beta R_{p}\right)$$

(20) $$\begin{array}{c} E_{k}^{FW}\left(t\right)=0 \end{array}$$

Among them, $A_{G\left(i\right)}$, $A_{E\left(j\right)}$ and $A_{p\left(k\right)}$ are the ith government agent, the jth reclaimed water agent, and the kth public agent respectively. x(t)、 z(t)、 z(t) represent the proportion of individuals in the population who adopt strategy PR, strategy DI, and strategy RW at time t, respectively.

Strategy learning rules are that the agent judges whether the current strategy is optimal by comparing the actual profits F(t) with the expected benefits E(t) of different strategies. And then determine the strategy selection in the t+1 period. The learning strategy rule of the local government agent is shown in equation (21):

(21) $$\begin{array}{c} S_{G\left(i\right)}\left(t+1\right)=\left\{ \begin{array}{c} 1-S_{G\left(i\right)}\left(t\right),S_{G\left(i\right)}\left(t\right)=1\;and\;E_{i}^{NR}\left(t\right)>\max\left\{\;E_{i}^{\mathrm{PR}}\left(t\right),F_{i}^{G}\left(t\right)\right\}\\ \;or\;S_{G\left(i\right)}\left(t\right)=0\;and\;E_{i}^{\mathrm{PR}}\left(t\right)>\max\left\{\;E_{i}^{NR}\left(t\right),F_{i}^{G}\left(t\right)\right\}\\ S_{G\left(i\right)}\left(t\right),others \end{array}\right. \end{array}$$

Similarly, the learning strategy rules of reclaimed water enterprise agent and the public agent are shown in equation (22), (23):

(22) $$\begin{array}{c} S_{E\left(j\right)}\left(t+1\right)=\left\{ \begin{array}{c} 1-S_{E\left(j\right)}\left(t\right),S_{E\left(j\right)}\left(t\right)=1\;and\;E_{j}^{DI}\left(t\right)>\max\left\{\;E_{j}^{CI}\left(t\right),F_{j}^{E}\left(t\right)\right\}\\ \;or\;S_{E\left(j\right)}\left(t\right)=0\;and\;E_{j}^{CI}\left(t\right)>\max\left\{\;E_{j}^{DI}\left(t\right),F_{j}^{E}\left(t\right)\right\}\\ S_{E\left(j\right)}\left(t\right),others \end{array}\right. \end{array}$$

(23) $$\begin{array}{c} S_{p\left(k\right)}\left(t+1\right)=\left\{ \begin{array}{c} {1-S}_{p\left(k\right)}\left(t\right),{\;S}_{p\left(k\right)}\left(t\right)=1\;and\;E_{k}^{FW}\left(t\right)>\max\left\{\;E_{k}^{RW}\left(t\right),F_{k}^{p}\left(t\right)\right\}\\ \;or\;{\;S}_{p\left(k\right)}\left(t\right)=0\;and\;E_{k}^{RW}\left(t\right)>\max\left\{\;E_{k}^{FW}\left(t\right),F_{k}^{p}\left(t\right)\right\}\\ S_{p\left(k\right)}\left(t\right),others \end{array}\right. \end{array}$$

We introduced a random interference term based on bounded rationality and assumed that the estimation results have an error of 0–30 % relative to the statistical outcomes, the error terms ϑ,μ,σ are obeyed in U(−0.15,0.15). Hence, x(t)、 z(t)、 z(t) are presented in equation (24)–(26).

(24) $$\begin{array}{c} x\left(t\right)=\left[NumA_{G}^{\mathrm{PR}}/TotalA_{G}\right]\left(0.85+\vartheta \right) \end{array}$$

(25) $$\begin{array}{c} y\left(t\right)=\left[NumA_{E}^{DI}/TotalA_{E}\right]\left(0.85+\mu \right) \end{array}$$

(26) $$\begin{array}{c} z\left(t\right)=\left[NumA_{p}^{RW}/TotalA_{p}\right]\left(0.85+\sigma \right) \end{array}$$

## 3 Simulation experiments

We used the NetLogo simulation platform to analyze the evolutionary game process in a two-dimensional square lattice. For a more intuitive comparison and to prevent excessive matching time for the central agent, the population sizes of the local government, reclaimed water enterprises, and the public are uniformly set at 500 (excluding scale effects). Moreover, we conducted 20 independent calculations on the above algorithms to reduce the interference of random factors. Referring to Wang et al. [9], the parameter setting is $S=({CB}_{g},C_{g},EB_{g},P_{g},B_{e},R_{e},C_{e},H_{e},S_{p},B_{p},R_{p},L_{p},\alpha \text{,}$.

β,γ). The specific parameter values were sequentially set as S=(14.1,39.3,10,20,3.2,3,2,5,5.5,6,.

2.5,5.8,0.3,0.5,0.2).

(1) Impact of subsidy intensity β on behavior strategies under different economic development levels

**Fig. 4:**
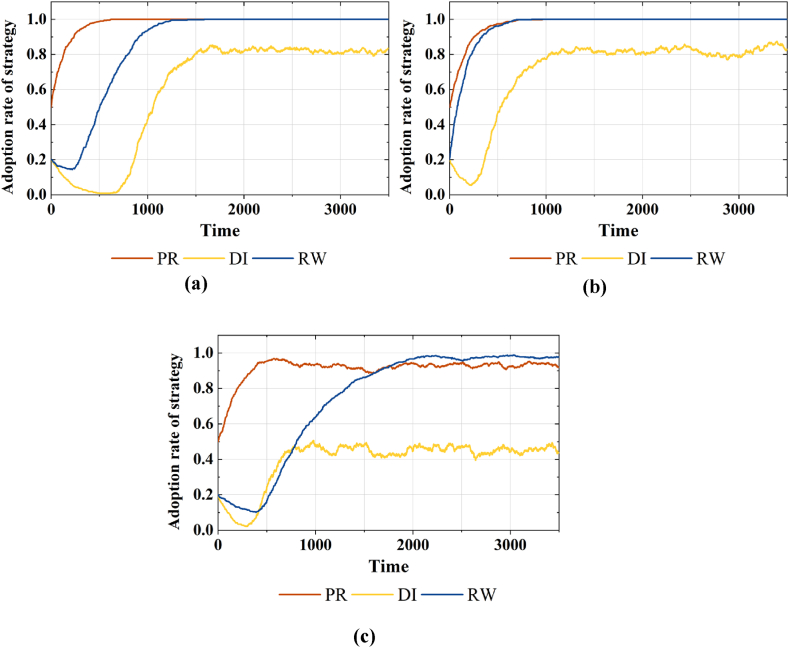
Strategy choices of various agents considering different β in areas with average economic development. (a)Adoption rates of strategy PR, DI and RW under β=0.1**, (b)**β=0.5**, and (c)**β=0.8.

**Fig. 5:**
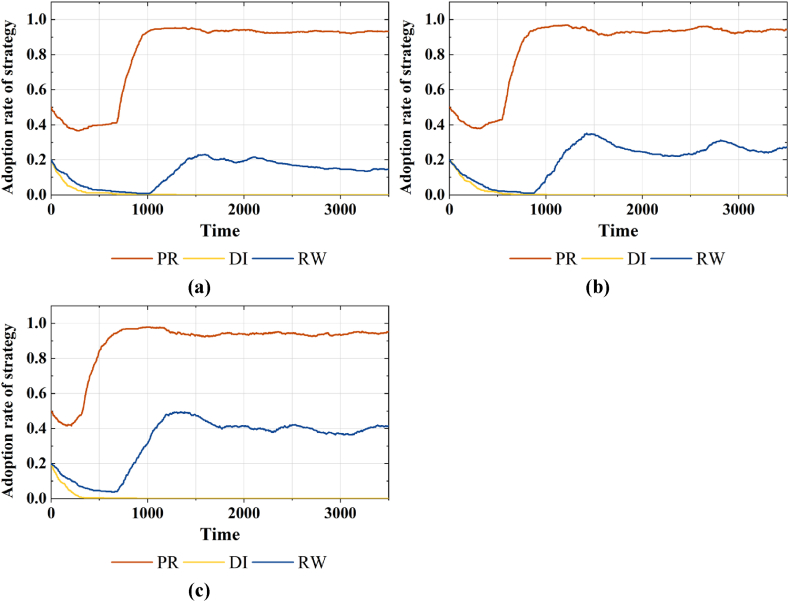
Strategy choices of various agents considering different β in areas with higher economic development. (a)Adoption rates of strategy PR, DI and RW under β=0.1**, (b)**β=0.5**, and (c)**β=0.8.

The regional development level influences the formulation of subsidy policy objectives and, at the same time, affects the sensitivity of the local public to the reclaimed water price. Therefore, in investigating the impact of subsidy intensity on behavior strategies, it is essential to consider the heterogeneity in regional economic development levels. Given the positive correlation between the regional economic development level and the local government's capacity to bear regulatory costs [45], we set that areas with higher economic development levels can take 80 % or more of the regulatory costs. That is, when ε≥0.8, the study area is with a higher level of economic development, and when ε<0.8, the study area is with an average. In addition, referring to Cao et al. [46], the subsidy intensity β is divided into three scenarios, i.e., β=0.1, 0.5 and 0.8, which represent conservative, moderate and radical subsidy. The 2*3 sets simulation results are shown in Fig. 4 and Fig. 5.

In Fig. 4(a)–(c), it can be seen that in regions with average economic development level, the increased subsidy intensity markedly elevates the proportion of the public population opting for reclaimed water strategies. But as the subsidy intensity further increases and exceeds 0.8, there is a reduction in the proportions of strategies PR and DI, and a fluctuating decline in the proportion of strategy RW. This change indicates an inverted U-shaped relationship between subsidy intensity and the public's willingness to use reclaimed water. For further study, in Fig. 5(a)–(c), it can be seen that in regions with higher economic development level, an increase in subsidy intensity can elevate the proportion of strategy RW in the public group, but its impact is insignificant.

(2) The impact of varying subsidy and supervision combinations on behavior strategies

**Fig. 6:**
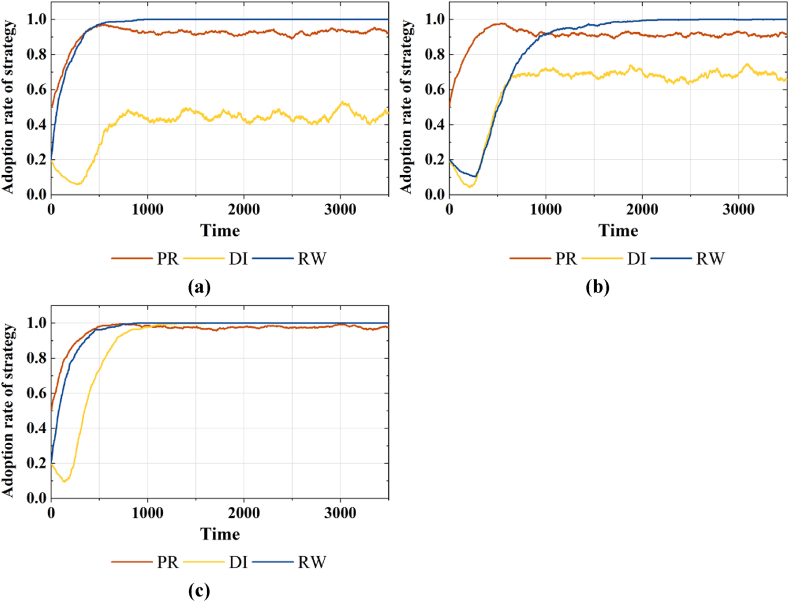
Strategy choices of various agents considering radical subsidy with different γ in areas with average economic development. (a)Adoption rates of strategy PR, DI and RW under β=0.8**with**γ=0.2**, (b)**γ=0.5**, and (c)**γ=0.9.

Similarly, referring to Cao et al. [46], the supervision intensity γ is divided into three scenarios, i.e., γ=0.2, 0.5 and 0.9, which represent light, moderate and high supervision. By exploring the impact of subsidy intensity on behavior strategies, we found that radical subsidy in regions with average economic development inhibits the demand for reclaimed water. Upon this simulation analysis, we set three combinations, {radical subsidy and light supervision, radical subsidy and moderate supervision, radical subsidy and high supervision}, to investigate further the influence of diverse supervision intensity on strategic choices under radical subsidy. The simulation results are illustrated in Fig. 6. The supervision intensity can enhance the proportion of strategy RW in the public group, and accelerates the convergence of this proportion to 1. Moreover, we observe that only under high supervision do government agents exclusively opt for strategy PR and maintain stability.

**Fig. 7:**
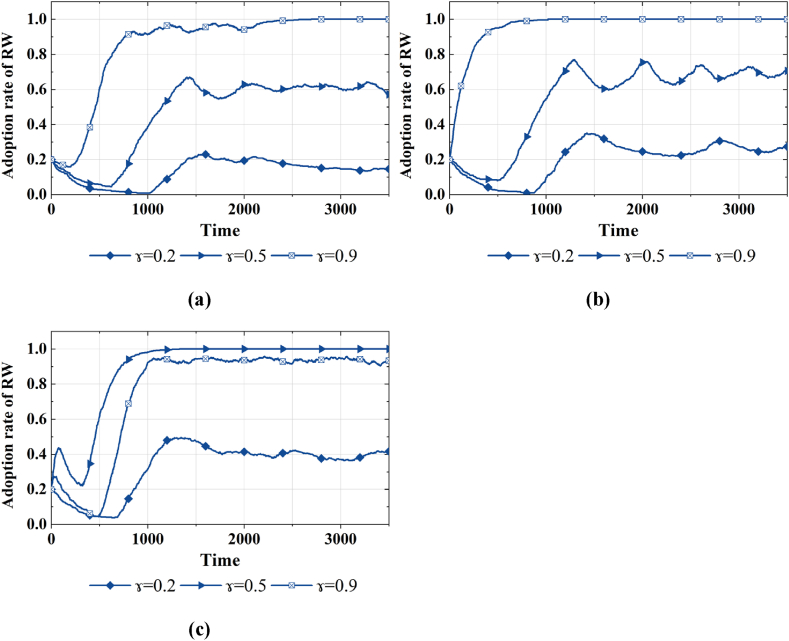
Strategy choices of the public agents considering different β and γ combinations in areas with higher economic development. (a) Adoption rates of strategy RW under a combination of γ=0.2,0.5,0.9 and β=0.1**, (b)**β=0.5**, and (c)**β=0.8.

By exploring the influence of subsidy intensity on agent behavior in areas with higher economic development levels, we found that the incentives of subsidies on reclaimed water utilization are not significant. Consequently, we further investigate the evolution of the public's willingness to use reclaimed water under different combinations of subsidy and regulatory intensities in this region. Simulation results are depicted in Fig. 7. A vertical observation of Fig. 7(a) and (b) reveals that under conservative and moderate subsidy, an increase in regulatory intensity can enhance the proportion of strategy RW in the public group. That is, the supervision of water quality information disclosure can positively modulate the incentives of price subsidies. However, upon further observation of Fig. 7(c), we find that the radical subsidy combined with the high supervision could decrease the proportion of strategy RW. This suggests that when supervision intensity exceeds 0.9, this positive modulation of supervision policy has a diminishing marginal effect.

## 4 Discussion

In the simulation experiment, we observed an inverted U-shaped relationship between subsidy policies and the public's willingness to use reclaimed water in regions with average economic development, with a threshold of 0.8. Specifically, a subsidy intensity exceeding 0.8 will lead to a fluctuating decrease in the willingness to use reclaimed water (As shown in Fig. 4). Investigate its reason, in regions with average economic development, the public's willingness to use reclaimed water is notably susceptible to price. And the subsidy policy can amplify the cost advantage, enhancing the demand for reclaimed water to a certain extent. However, the subsidy policy entails substantial financial expenditure. Prolonged implementation will exert economic pressure on the government and augment revenue risks, prompting recurrent government strategy adjustments. The recurrent oscillations in government strategies directly cause fluctuations in the purchasing cost, subsequently affecting the demand in the reclaimed water market. Simultaneously, enterprises may contemplate reducing their information disclosure spending due to the uncertainty of vested interests caused by government strategies. Further research has shown that, under radical subsidies, diminishing incentive effects can be mitigated only by implementing high supervision on water quality information disclosure (As shown in Fig. 5). Investigate its reason, a smaller revenue base of penalty provides only a limited cost compensation transfer to the government. Prolonged information concealment also amplifies the government's challenge in acquiring factual product information over the long term. Therefore, the government's willingness to positively regulate will decrease.

Further analysis of simulation experiments reveals that in regions with higher levels of economic development, the impact of subsidy policies on promoting the public's willingness to use reclaimed water is not significant. Fortunately, water quality information disclosure supervision positively moderates the incentive effects of subsidy policies (As shown in Fig. 6(a)–(b)). This further underscores the higher sensitivity of the public in this region to water quality information compared to price factors, and the finding is consistent with that of a previous study [19]. However, diverging from prior studies [17,19], our study conducted additional experiments with varying combinations of supervision intensity and subsidy levels (As shown in Fig. 6(c)). The results indicated that, under radical subsidies, high supervision could suppress the inclination to utilize reclaimed water. Investigate its reason, excessive regulation and intervention can erode market stability. On one hand, heightened supervision can breed resistance among producers, fostering rent-seeking behavior. On the other hand, enterprises may seek to transfer breach costs to end-users by increasing the reclaimed water price, potentially diminishing consumer surplus and impeding market development.

## 5 Conclusions and policy implications

The multi-agent-based simulation model driven by evolutionary game theory was constructed with the local government, reclaimed water enterprises, and the public as participants. Under the framework of ‘external environment-perceived value-utilization intention,’ from micro to macro perspective, we explored the composite effects of government subsidy policies and water quality information disclosure supervision policies on reclaimed water utilization. The conclusions are as follows.

(1) This study has elucidated a discernible threshold effect concerning subsidy policies and the public's inclination to embrace reclaimed water in regions with average economic development. The observed relationship manifests as an inverted U-shaped curve, signifying an optimal value for the promotional impact. Specifically, when the subsidy intensity is below 0.8, there is a positive correlation between subsidy intensity and the public's willingness. However, surpassing the 0.8 threshold in subsidy intensity reveals the unsustainable nature of radical subsidy approaches, intensifying the volatility risk associated with the cost of reclaimed water utilization and consequently diminishing the willingness to adopt reclaimed water.
(2) In regions with average economic development, supervision of information disclosure behavior can mitigate the diminishing incentivizing effect under radical subsidies. However, the assisting effect of different supervision intensities exhibits heterogeneity. Specifically, when the supervision intensity increases to 0.9, the public's willingness to use reclaimed water under radical subsidies, while moderately enhanced, remains relatively subdued, accompanied by an ambiguous preference for water sources. Conversely, when the intensity surpasses 0.9, the public exhibits a robust intention to utilize reclaimed water under radical subsidies.
(3) The study further reveals that subsidy policies alone do not significantly enhance the public's willingness to use reclaimed water in regions with higher economic development. However, when complemented by water quality information disclosure supervision, the incentivizing effect becomes pronounced. Moreover, the interplay between subsidy and supervision policies manifests different chain reactions under various intensities. Among these, the combination of moderate subsidy and high supervision yields the most optimal outcome.

Based on these results, three policy implications have been proposed to governments to promote reclaimed water development.

Firstly, according to the findings, determining the optimal subsidy intensity (below 0.8) is crucial to significantly boosting the willingness to use reclaimed water in regions with average economic development. The government should set subsidies within this range to maximize their effectiveness. Additionally, to address limited local regulatory funding capacity, policies encouraging private sector investment in reclaimed water production and expanding funding sources should be considered.

Secondly, in regions with higher economic development, where public sensitivity to subsidies is lower, the government should prioritize optimizing the supervision mechanisms for water quality information disclosure. The study suggests that the most effective approach to enhance willingness to use reclaimed water in such areas is a policy combination of moderate subsidies and high supervision. Therefore, the government should consider overlaying supervision and subsidy policies, giving preference to schemes with moderate subsidy intensity while maximizing the strength of water quality information disclosure supervision.

Finally, dynamic, differentiated subsidies and supervision should be implemented to enhance policy precision. On the one hand, strengthening the evaluation process is essential, allowing for dynamic adjustments to subsidy and supervision intensities based on changes in public willingness to use reclaimed water and the status of water quality information disclosure. On the other hand, factors such as the scale of reclaimed water facilities, water quality grades, and geographical characteristics should be considered when implementing these policies.

We acknowledge the limitations of the evolutionary game model, akin to similar agent behavior analyses, which simplifies social states, which may lead to a gap between theoretical and practical applications. While we have introduced a random error term to reduce discrepancies, future research endeavors should explore additional methods to minimize such errors. Furthermore, our study primarily focuses on systemic policy effects at a regional level. Future investigations will benefit from segmenting users into specific categories, thereby enabling a more nuanced exploration of policy responses across different user groups.

## Funding

We appreciate the editors and anonymous reviewers for their constructive comments. This work was supported by the Postgraduate Research & Practice Innovation Program of 10.13039/501100002949Jiangsu Province under Grant No. KYCX23_0645, and the National Nature Science Foundation of China under Grant No. 42271303.

## Ethics statement

Review and/or approval by an ethics committee was not needed for this study because our work does not involve any use of animal subjects.

Informed consent was not required for this study because our work does not involve any interaction with or observation of people.

## Data availability statement

The data associated with our study has not been deposited into a publicly available repository, but data will be made available on request.

## CRediT authorship contribution statement

**Wei Wang:** Writing – original draft, Funding acquisition. **Fengping Wu:** Supervision, Funding acquisition.

## Declaration of competing interest

The authors declare that they have no known competing financial interests or personal relationships that could have appeared to influence the work reported in this paper.
